# Complete Genomic DNA Sequence of the East Asian Spotted Fever Disease Agent *Rickettsia japonica*

**DOI:** 10.1371/journal.pone.0071861

**Published:** 2013-09-09

**Authors:** Minenosuke Matsutani, Motohiko Ogawa, Naohisa Takaoka, Nozomu Hanaoka, Hidehiro Toh, Atsushi Yamashita, Kenshiro Oshima, Hideki Hirakawa, Satoru Kuhara, Harumi Suzuki, Masahira Hattori, Toshio Kishimoto, Shuji Ando, Yoshinao Azuma, Mutsunori Shirai

**Affiliations:** 1 Department of Microbiology and Immunology, Yamaguchi University School of Medicine, Ube, Yamaguchi, Japan; 2 Department of Virology 1, National Institute of Infectious Diseases, Tokyo, Japan; 3 Kitasato Institute for Life Sciences, Kitasato University, Sagamihara, Kanagawa, Japan; 4 Genetic Resources Technology, Faculty of Agriculture, Kyushu University, Fukuoka, Japan; 5 Department of Science and Technology on Food Safety, Kinki University BOST, Kinokawa, Wakayama, Japan; University of Poitiers, France

## Abstract

*Rickettsia japonica* is an obligate intracellular alphaproteobacteria that causes tick-borne Japanese spotted fever, which has spread throughout East Asia. We determined the complete genomic DNA sequence of *R. japonica* type strain YH (VR-1363), which consists of 1,283,087 base pairs (bp) and 971 protein-coding genes. Comparison of the genomic DNA sequence of *R. japonica* with other rickettsiae in the public databases showed that 2 regions (4,323 and 216 bp) were conserved in a very narrow range of *Rickettsia* species, and the shorter one was inserted in, and disrupted, a preexisting open reading frame (ORF). While it is unknown how the DNA sequences were acquired in *R. japonica* genomes, it may be a useful signature for the diagnosis of *Rickettsia* species. Instead of the species-specific inserted DNA sequences, rickettsial genomes contain Rickettsia-specific palindromic elements (RPEs), which are also capable of locating in preexisting ORFs. Precise alignments of protein and DNA sequences involving RPEs showed that when a gene contains an inserted DNA sequence, each rickettsial ortholog carried an inserted DNA sequence at the same locus. The sequence, ATGAC, was shown to be highly frequent and thus characteristic in certain RPEs (RPE-4, RPE-6, and RPE-7). This finding implies that RPE-4, RPE-6, and RPE-7 were derived from a common inserted DNA sequence.

## Introduction

*Rickettsia japonica* is an obligate intracellular bacterium isolated from ticks, wild animals, and human patients [1]. *R. japonica* was first identified as a causative agent of Japanese spotted fever [2] and has been isolated from patients in South Korea, the Philippines, and Thailand only in the past decade [3]–[5]. For rapid and accurate diagnosis of rickettsial infections, a new diagnostic assay of *R. japonica* using a species specific DNA sequence was proposed on the basis of a comparative genome analysis of rickettsiae isolated from Asian countries [6].

*Rickettsia* species have been divided into at least 3 groups on the basis of phylogenetic relatedness, i.e., spotted fever group (SFG), typhus group (TG), and ancestral group (AG) [7]. *R. japonica* is categorized into SFG as well as *R. rickettsii* and *R. conorii*. The TG includes epidemic and endemic typhus *Rickettsia*, *R. prowazekii* and *R. typhi*, respectively. *R. canadensis* and *R. bellii* belong to the AG [8]–[11]. This simplistic taxonomical assignment is not always suitable for classifications based on ecological and clinical traits and thus remains controversial to date [12].

Ogata and coworkers showed the existence of highly frequent repeat sequences of 100–150 bp in length in *Rickettsia* genomes called *Rickettsia* palindromic elements (RPEs) [13]. RPEs have been identified in all *Rickettsia* and *Wolbachia* species sequenced so far [14]. While other bacterial palindromic repeats are mostly located within non-coding regions, certain RPEs have been found in preexisting open reading frames (ORFs) without destroying the frames. In particular, RPE-1, RPE-2, and RPE-3 are frequently observed in ORFs [13], [15], [16].

In the present study, to develop diagnostic tools and investigate rickettsial genome evolution, we completed the genomic DNA sequence of *R. japonica* type strain YH (VR-1363), and showed common features of inserted DNA sequences involving RPE-4, RPE-6, and RPE-7.

## Materials and Methods

### Genomic DNA sequencing

Genome DNA sequencing of *R. japonica* was performed using the same methods as those previously described [17]. Briefly, *R. japonica* YH (VR-1363) was originally isolated from a specimen of a Japanese patients with spotted fever [18]. *R. japonica* was cultured with Vero cells, and the bacterial cells for genomic DNA preparation were purified from the infected host cells using Dounce homogenization, differential centrifugation, and Percoll density gradient centrifugation. DNA sequencing was performed using Sanger method with a 3730xl sequencer (Life Technologies) though all sequencing processes, i.e., random sequencing and gap closing stages. Whole obtained sequence data (17,664 reads) after removing sequences of vector and sequencing adaptor used here were analyzed by BLASTN against human genome database to remove contamination sequences derived from Vero cells. As a result, shotgun data were obtained with 15,502 reads, which gave 10.0×coverage. The sequences were assembled into 256 contigs using the Phred/Phrap/Consed package software [19]. Additional 1,162 sequence reads were determined by direct sequencing of PCR products to close gaps and to reconfirm sequences of regions with low qualities.

### ORF prediction and annotation

Protein-coding genes were first predicted using a pool of open reading frame (ORF) candidates indicated by 3 programs GenomeGambler ver. 1.51 [20], GeneHacker plus [21], and Glimmer 2.0 [22]. If multiple translation start sites were suggested for an ORF, manual inspection was performed to select the most probable start site on the basis of the package with the nearest upstream ORF, similarities to homologous genes, and the predicted Shine–Dalgarno sequence. Annotation for each ORF was performed using a program BLASTP [23] against genes in *R. conorii* genome [24], the NCBI non-redundant protein database and COGs [25]. Lastly, protein-coding genes were manually identified on the basis of the combination of the results of ORF prediction and annotation. All new sequence data of *R. japonica* YH VR-1363 have been deposited in DDBJ/EMBL/GenBank, the accession number: AP011533.

### RPE analyses

For identification of RPEs in *R. japonica* genome DNA sequences, we used a sequence similarity search program, HMMER3 [26], based on hidden Markov models, and the previously identified RPE sequences [24] (http://igs-server.cnrs-mrs.fr/mgdb/Rickettsia/) as a training data set. Rickettsial orthologs of the *R. japonica* genes with RPE-4, RPE-6, and RPE-7 were identified by reciprocal best hit using BLASTP [23] (filtering expectation values: a cut off E-value above 10^−10^ and a sequence overlap cut off below 70%) with considering synteny around the genes. Herein, we showed data using 6 *Rickettsia* species, *R. conorii* [13], *R. africae* [27], *R. felis* [28], *R. prowazekii* [29], *R. typhi* [30], and *R. japonica*. DNA sequences of RPE-4, RPE-6, and RPE-7, and the boundary regions of the *Rickettsia* orthologous genes were aligned with corresponding regions of non-rickettsial orthologous genes using the ClustalW program [31]. Highly frequent sequences were identified from the RPEs by MEME version 4.0.0, which is part of the Meta-MEME package [32]. Motif sequences were illustrated using a sequence LOGO format (http://weblogo.berkeley.edu) [33].

The location of amino acid fragments encoded by inserted DNA sequences were analyzed on the basis of homology structure modeling of rickettsial proteins using *Escherichia coli* DNA polymerase III alpha chain, *Bacillus subtilis* tRNA ribosyltransferase-isomerase, and *Thermus thermophilus* phenylalanyl-tRNA synthetase subunit beta (for which the Protein Data Bank Identifiers (PDB IDs) are 2hnhA, 1yy3A, and 1b70B, respectively) [34].

### Unique region analyses

Comparison of the genomic DNA sequence of *R. japonica* YH VR-1363 with other 27 rickettsiae in the public databases (listed in Table S1) were carried out using BLASTN (a cut-off E-value >e^−5^) to figure out *R. japonica* unique regions. Low similarity regions shown from the first screening were re-analyzed using BLASTN (a cut-off E-value >e^−5^) to assign the unique regions.

## Results and Discussion

### General features of the *R. japonica* genome

**Figure 1:**
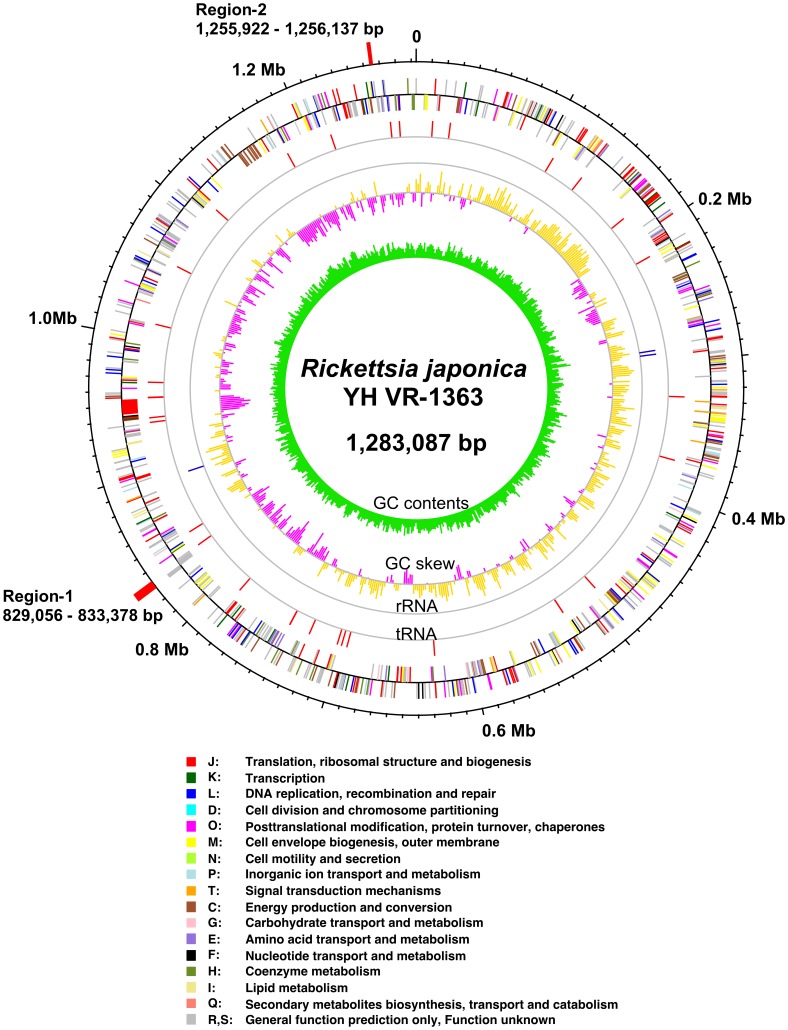
Circular exhibition of the *Rickettsia japonica* YH genome. The outermost scale is marked for nucleic acid position in Mbp, and Region-1 and -2 (red). From the outside (track 1), gene positions and directions (clockwise on the outside and anti-clockwise on the inside) of each gene were classified and colored based on COGs [45]. Track 2 and 3: tRNA (red) and rRNA (blue), respectively. Track 4: GC skew, outside yellow and inside purple indicate values >0 and values <0 as calculated by (G−C/G+C) [46]. Track 5: innermost, GC contents. Based on the cumulus of the GC skew values, accompanied by other *Richettsia* genomes, a hypothetical origin, shown as *ori*, was determined and the base numbers were counted from the origin.

The genomic DNA of *R. japonica* strain YH consists of a 1,283,087-bp circular chromosome with 32.4% GC content, and no plasmids were identified (Fig. 1). A total of 971 protein-coding genes were identified in the genome, and those ORFs covered 80.1% of the chromosome. Putative functions were assigned to 706 genes. The genomes of *Rickettsia* species belonging to the SFG are well known to contain numerous pseudogenes, and many of their orthologs in *R. felis* are still intact [28], [35]. We identified 202 pseudogenes in the *R. japonica* genome, and the 144 orthologs in *R. felis* are unbroken. The *R. japonica* genome includes all known virulence factors conserved among SFG rickettsiae, such as rOmpA (which functions as the initial adhesion and is conserved in SFG [36]), rOmpB (which plays an important role for recognition and invasion, and is conserved widely in rickettsiae [37]), phospholipase A_2_ (which has been proposed to mediate escape from phagosomes [38]), InvA and RickA (which mediate actin polymerization and motility [39]), and 3 hemolysins (i.e., hemolysin A, hemolysin C, and hemolysin-like protein).

Recently the draft genome sequence of *R. japonica* type strain YH (VR-1336) have been published [40]. There are no differences between DNA sequences of 16S rRNA genes (1,508 bp) of *R. japonica* YH (VR-1336) and *R. japonica* YH (VR-1363), and 23S-5S rRNA gene operons (3,151 bp) as well (a phylogenetic tree was shown in Figure S1A). In contrary, amino acid sequences of the *R. japonica* VR-1336 gene products are relatively diverse from *R. japonica* YH VR-1363, resulted in presentation of a different phylogenetic tree from one based on rRNA genes (Figure S1B). The precise taxonomical classification and nomenclatural scheme of *Rickettsia* species should be clarified in the future.

### Unique traits of the *R. japonica* genome

Comparison of the genomic DNA sequence of *R. japonica* YH VR-1363 with the sequences of other 27 rickettsiae in the public databases (Table S1) showed that 2 regions (Region-1 and Region-2) were unique to only a few rickettsiae including *R. japonica* (a cut off E-value above e^−5^) (Fig. 1). The Region-1, which was located at 829,056–833,378 bp in *R. japonica* genome, was conserved only in *R. japonica* and *R. heilongjiangensis* [41], and Region-2 at 1,255,922–1,256,137 bp was conserved in *R. japonica*, *R. heilongjiangensis*, *Candidatus Rickettsia amblyommii* (Accession number: CP003334) and *Rickettsia montanensis* (CP003340). Polymerase chain reaction (PCR) analysis clarified that Region-2 was conserved among all 5 strains of *R. japonica* (i.e., YH, DT-1, FLA-1, HH-8, and HH-9) [6]. Region-1 consists of 12 genes encoding 3 integrases (RJP_0637-RJP_0639), 2 protein kinases (RJP_0642-RJP_0643), and 7 hypothetical proteins (RJP_0640, RJP_0641, RJP_0644-RJP_0648). Region-2 carries a hypothetical protein (RJP_0988). Similarities of all 13 genes to other organisms except rickettsiae were rather low, and the organisms carrying these similar genes varied widely from alphaproteobacteria to plants and animals. For instance, amino acid sequences of the integrases are partially similar to integrases from *R. belli*, *R. felis*, *R. massiliae*, and *Orientia tsutsugamushi*, but not to integrases from *Rickettsia* species closely related in the SFG. Interestingly, the hypothetical protein in Region-2 contains ankyrin repeats and is partially similar to mammalian proteins, such as *Tupaia chinensis* myosin-XVI (ELW47454) with 50% identity out of 32 aa of RJP_0988. Lastly, since the DNA sequences of Region-1 and Region-2 are unique to only a few *Rickettsia* species and there are some differences in DNA sequences of the species, it is very useful to recognize and distinguish *Rickettsia* species [6].

The *R. japonica* genome shows genome-wide synteny against SFG rickettsial genomes, while there are 2 genome rearrangements occurred between 668 and 754 kb, and 851 and 939 kb in the *R. japonica* YH genome (Figure S2). These regions are thought to be hotspots for genome rearrangements of *Rickettsia* [30] since frequent rearrangements were observed.

### Common features of RPE-4, RPE-6, and RPE-7

RPEs in the genes of *R. japonica* were identified by hidden Markov models. The data showed that the *R. japonica* genes with RPEs and their inserted positions were conserved in SFG *Rickettsia* genome evolution. In the case of genes with RPE-1, RPE-2, and RPE-3, rickettsial orthologs of the genes contained the same group of RPEs at similar sites [13]. In contrast, in the case of genes carrying RPE-4, RPE-6, and RPE-7, no typical RPEs were observed in some rickettsial orthologs; or they contained different groups of RPEs located near to each other but at different positions (Table 1). It seems that there are at least 2 possibilities. It could be that the insertion sites for RPE-4, RPE-6, and RPE-7 are hotspots for insertions of different origins. The other possibility is that RPE-4, RPE-6, and RPE-7 originated from the same inserted DNA sequence but widely diverged thereafter.

**Table 1:** List of genes with RPE-4, RPE-6 and RPE-7 and insertion lengths in *R. japonica*.

*R. japonica*			*R. conorii*	*R. africae*	*R. felis*	*R. prowazekii*	*R. typhi*	
Gene ID	RPE length	RPE group	RPE group	RPE group	RPE group	RPE group	RPE group	*Product*
RJP_0284	84	RPE-4	RPE-4	RPE-4	ND	ND	ND	multisubunit Na+/H+ antiporter, mnhB subunit
RJP_0560	142	RPE-4	RPE-4	RPE-4	RPE-4	ND	ND	putative aminomethyltransferase
RJP_0434	98	RPE-4	RPE-4	RPE-4	RPE-6	ND	ND	recB family exonuclease
RJP_0891	114	RPE-4	RPE-4	RPE-4	RPE-6	ND	ND	DNA polymerase III subunit alpha
RJP_0222	115	RPE-4	RPE-4	RPE-4	RPE-7	ND	ND	tRNA ribosyltransferase-isomerase
RJP_0300	90	RPE-6	RPE-6	RPE-6	RPE-7	ND	ND	NADH dehydrogenase subunit N
RJP_0790	192	RPE-4	RPE-4	RPE-4	RPE-7	ND	ND	proline/betaine transporter
RJP_0384	137	RPE-4	RPE-4	RPE-4	RPE-7	ND	RPE-6	putative lipid A core - O-antigen ligase
RJP_0202	150	RPE-6	RPE-6	RPE-6	RPE-6	ND	RPE-4	bifunctional penicillin-binding protein 1C
RJP_0524	96	RPE-6	RPE-6	RPE-6	RPE-4	ND	ND	biotin-(acetyl-CoA carboxylase) ligase
RJP_0634	101	RPE-6	RPE-6	RPE-6	RPE-4	ND	ND	excinuclease ABC subunit C
RJP_0964	108	RPE-6	RPE-6	RPE-6	RPE-7	ND	ND	tRNA (guanine-N(7)-)-methyltransferase
RJP_0013	68	RPE-7	RPE-7	RPE-7	RPE-7	ND	RPE-7	ABC transporter substrate binding protein
RJP_0722	144	RPE-7	RPE-7	RPE-7	RPE-6	ND	ND	ATP-dependent DNA helicase
RJP_0124	114	RPE-7	RPE-7	RPE-7	RPE-6	ND	ND	putative nucleoside-diphosphate-sugar epimerase
RJP_0449	108	RPE-7	RPE-7	RPE-7	RPE-7	ND	ND	phenylalanyl-tRNA synthetase beta subunit
RJP_0691	144	RPE-7	RPE-7	RPE-7	RPE-7	ND	ND	isoleucyl-tRNA synthetase
RJP_0552	96	RPE-7	RPE-7	RPE-7	RPE-7	RPE-4	ND	soluble lytic murein transglycosylase

ND: typical RPE-4, RPE-6 and RPE-7 were not detected while orthologous genes and insertions exist.

**Figure 2:**
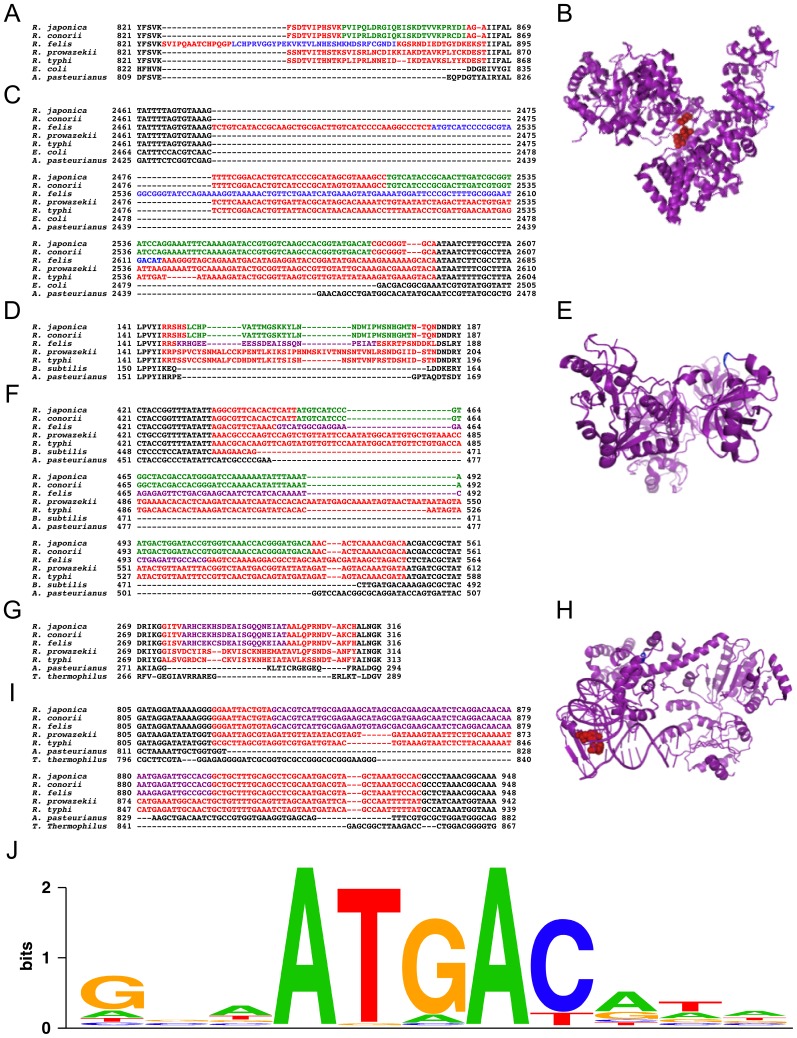
Multiple alignments of amino acid and DNA sequences, including inserted DNA sequences. Each panel shows A–C) DNA polymerase III alpha chain; D–F) tRNA ribosyltransferase-isomerase; G–I) phenylalanyl-tRNA synthetase subunit beta; A, D, G) amino acid alignments; B, E, H) structure models; C, F, I) DNA alignments. Letters in alignments indicate green, typical RPE-4s; blue, typical RPE-6s; purple, typical RPE-7s; red, extended RPE-4 regions; black, boundary regions of genes with inserted DNA sequences. Amino acids encoded by inserted DNA sequences were shown by red balls in the protein structures. Gene sequences were extracted from each genome sequence; *Rickettsia japonica* (this work); *R. conorii*, AE006914 [13]; *R. felis*, CP000053 [28]; *R. prowazekii*, AJ235269 [29]; *R. typhi*, AE017197 [30]; *Acetobacter pasteurianus* IFO 3283-01, AP011121 [47]. Protein Data Bank Identifiers (PDB IDs) for amino acid sequences of *Escherichia coli* DNA polymerase III alpha chain, *Bacillus subtilis* tRNA ribosyltransferase-isomerase, and *Thermus thermophilus* phenylalanyl-tRNA synthetase subunit beta are 2hnhA, 1yy3A, and 1b70B, respectively. J) The highly conserved short sequence in e-RPEs were illustrated using a sequence LOGO format [33].

To clarify the possibilities, we precisely aligned 90 RPE loci using amino acid and, thus, DNA sequences from 18 orthologs of 5 rickettsial genomes (3 ortholog examples are shown in Fig. 2) with orthologs from other bacteria. The result obviously indicated that all *Rickettsia* orthologs contained inserted DNA sequences at the same sites, but the insertions were extended from typical RPEs called extended RPE-4 (eRPE-4). eRPE-4 sequences were identified even in the orthologs without typical RPEs (Table 2 and Fig. 2). Location analysis of amino acid fragments encoded by eRPE-4s illustrated that the protein domains, including the extended regions, were located in solvent-exposed areas (Fig. 2), which was similar to RPE-1, RPE-2, and RPE-3 [15]. Analysis using MEME software [42] showed that ATGAC sequences, which were originally found as part of palindromic sequences in RPE-4, RPE-6, and RPE-7, were shown to be highly frequent not only in the typical RPEs but also in the extended region of eRPE-4s (Fig. 2J, Table 2). The frequencies of the ATGAC motif were significantly higher in eRPE-4 than in non-RPE regions (Table 2), and those in RPE-1, RPE-2, and RPE-3 were significantly lower than the frequencies in eRPE-4s and any other genome region. In addition, we found that biotin-(acetyl-CoA carboxylase) ligase genes of *Wolbachia* and *Rickettsia* retained *Wolbachia* palindromic elements (WPEs) [43] and eRPE-4 in the same region of each gene. Additionally, the frequency of the ATGAC motif in WPEs was high (data not shown). However, the possibility that the RPEs were inserted into certain hotspots is still not negligible. The observations implicated that RPE-4, RPE-6, and RPE-7 (and possibly WPEs) were derived from the same origin, which was different from the origins of RPE-1, RPE-2, and RPE-3. Thus, the initial insertion occurred before the divergence of Rickettsiaceae after the separation of Rickettsiaceae from alphaproteobacteria.

**Table 2:** Frequency of the ATGAC sequences in extended RPE-4s (eRPE-4s).

Regions	Category of eRPE-4		Length (bp)	Amount of ATGAC	Frequency (per kb)
eRPE-4	eRPE-4 including RPE-4	Total	2,243	35	15.6
		RPE-4	806	27	33.5
		Extended	1,437	8	5.6
	eRPE-4 including RPE-6	Total	1,805	35	19.4
		RPE-6	1,235	20	16.2
		Extended	570	15	26.3
	eRPE-4 including RPE-7	Total	2,291	33	14.4
		RPE-7	878	18	20.5
		Extended	1,413	15	10.6
	eRPE-4 without RPE-4,6,7	Extended	3,565	17	4.8
RPE-1,-2,-3,-5		24,452	3	0.1	
Other regions		1,248,731	2,311	1.9	
Whole genome of *R. japonica*		1,283,087	2,417	1.9	

ND: Not detected.

## Conclusions

An accurate diagnosis of rickettsial infections is desirable to distinguish these infections from other similar diseases such as leptospirosis and dengue fever, because rickettsioses is remediable by suitable antimicrobial therapy. In this study, we performed complete sequencing of the genomic DNA of *R. japonica*, which causes tick-borne Japanese spotted fever prevalent in East Asia [44]. Compared to other *Rickettsia* species, *R. japonica* YH (VR-1363) genome contains 2 unique regions, Region-1 and Region-2. Evolutional origins of these regions are not clear, but the DNA sequences will be a useful tool for the diagnosis of *Rickettsia* species [6]. Two rearrangement regions specific to *R. japonica* might be valuable for distinguishing *Rickettsia* species in SFG as well. The precise alignments of DNA sequences of genes involving RPE-4, RPE-6, and RPE-7 showed that insertions of these sequences could occur in the same loci of orthologous genes. Those observations imply that RPE-4, RPE-6, and RPE-7 diverged from a common origin and, thus, may be a valuable signature for the diagnosis of *Rickettsia* species.

## Supporting Information

Figure S1 **Neighbor-joining phylogenetic trees of SFG Rickettsias.** A) DNA sequences of 23s-5s rRNA gene operons were aligned with ClustalW [31] and the phylogenetic tree was constructed using Seaview4 [48]. B) Amino acid sequences produced by concatenation of 670 gene products shared with SFG *Rickettsia* were aligned with ClustalW [31] and the phylogenetic tree was constructed using MEGA5.05 [49]. SFG Rickettsias used here were *R. africae* ESF-5 (CP001612), *R. parkeri* Portsmouth (CP003341), *R. conorii* Malish 7 (AE006914), *R. slovaca* 13-B (CP002428), *R. peacockii* Rustic (CP001227), *R. philipii* 364D (CP003308), *R. rickettsii* Sheila Smith (CP000848), *R. heilongjiangensis* 054 (CP002912), *R. japonica* YH VR-1363 (AP011533), *R. japonica* YH VR-1336 (AMRT00000000), *R. rhipicephali* 3–7-female6-CWPP (CP003342), *R. massiliae* MTU5 (CP000683), *R. montanensis* OSU 85-930 (CP003340), and *R. felis* URRWXCal2 (CP000053).
(TIF)

Figure S2 **Dot-plot analyses of** ***R. japonica*** **with Rickettsiae.** Highly similar regions in genomic DNA sequences of *Rickettsia* species to *R. japonica* YH VR-1363 were bi-directionally shown by dot-plot analysis using GenomeMatcher [50]. Red and green spots indicate similar regions in forward- and complementary-strands of each genome sequence, respectively. Inverted and translocation positions were indicated by 4 vertical lines at 668, 754, 851 and 939 kb of *R. japonica* genome. Rickettsiae used here were *R. japonica* YH VR-1363 (AP011533), *R. conorii* Malish 7 (AE006914), *R. rickettsii* Sheila Smith (CP000848), *R. heilongjiangensis* 054 (CP002912), *R. massiliae* MTU5 (CP000683), *R. felis* URRWXCal2 (CP000053) and *R. prowazekii* str. Madrid E (AJ235269).
(TIF)

Table S1 ***Rickettsia*** **species for analyses.**
(DOC)
