# Supplementary figures and images for: Complete Genomic DNA Sequence of the East Asian Spotted Fever Disease Agent Rickettsia japonica
Source: PLoS One. 2013 Sep 9;8(9):e71861. doi: 10.1371/journal.pone.0071861 (PMC3767692; doi:10.1371/journal.pone.0071861)

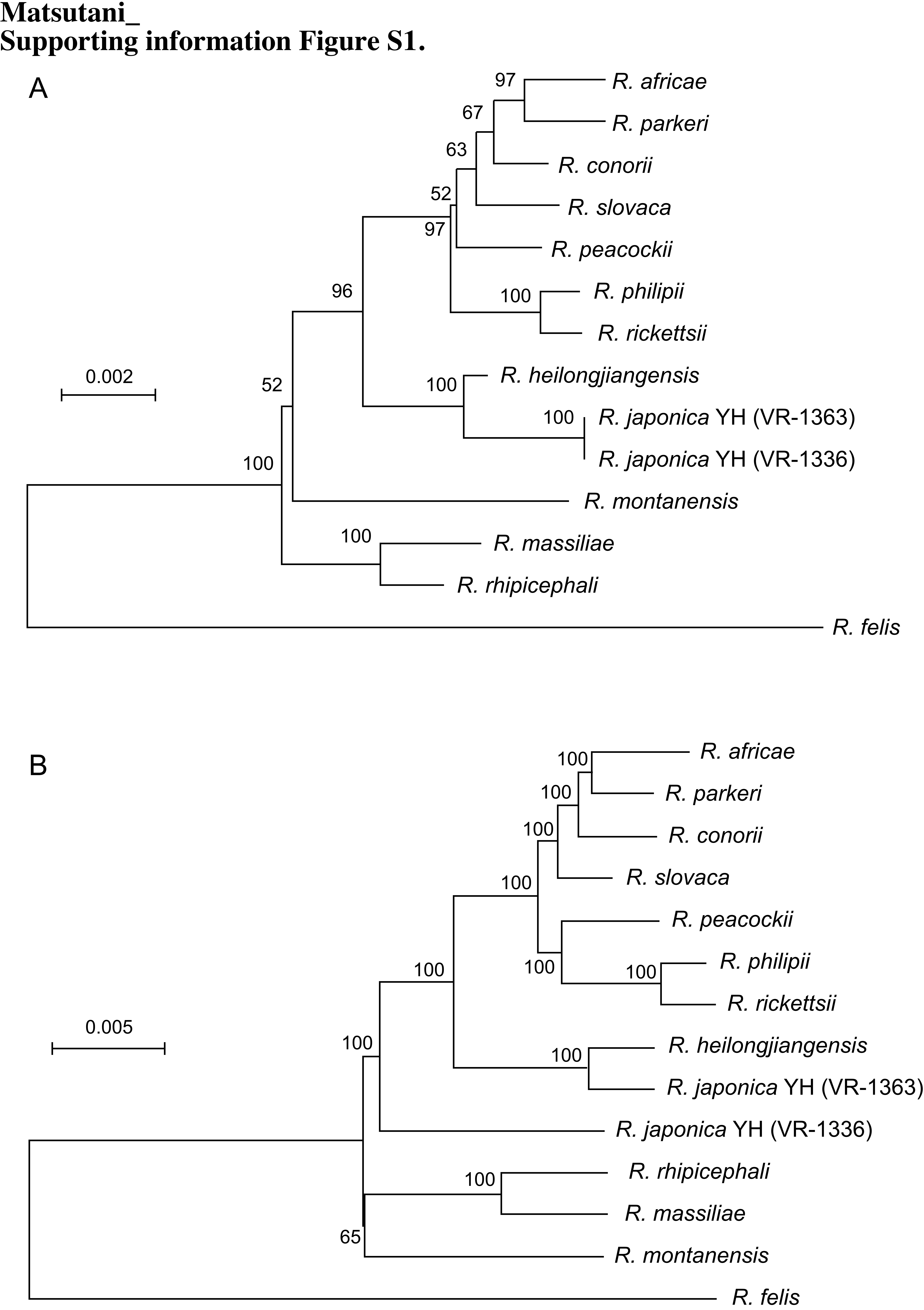

Supplement: Figure S1 — Neighbor-joining phylogenetic trees of SFG Rickettsias. A) DNA sequences of 23s-5s rRNA gene operons were aligned with ClustalW [31] and the phylogenetic tree was constructed using Seaview4 [48]. B) Amino acid sequences produced by concatenation of 670 gene products shared with SFG Rickettsia were aligned with ClustalW [31] and the phylogenetic tree was constructed using MEGA5.05 [49]. SFG Rickettsias used here were R. africae ESF-5 (CP001612), R. parkeri Portsmouth (CP003341), R. conorii Malish 7 (AE006914), R. slovaca 13-B (CP002428), R. peacockii Rustic (CP001227), R. philipii 364D (CP003308), R. rickettsii Sheila Smith (CP000848), R. heilongjiangensis 054 (CP002912), R. japonica YH VR-1363 (AP011533), R. japonica YH VR-1336 (AMRT00000000), R. rhipicephali 3–7-female6-CWPP (CP003342), R. massiliae MTU5 (CP000683), R. montanensis OSU 85-930 (CP003340), and R. felis URRWXCal2 (CP000053). (TIF) [file pone.0071861.s001.tif]

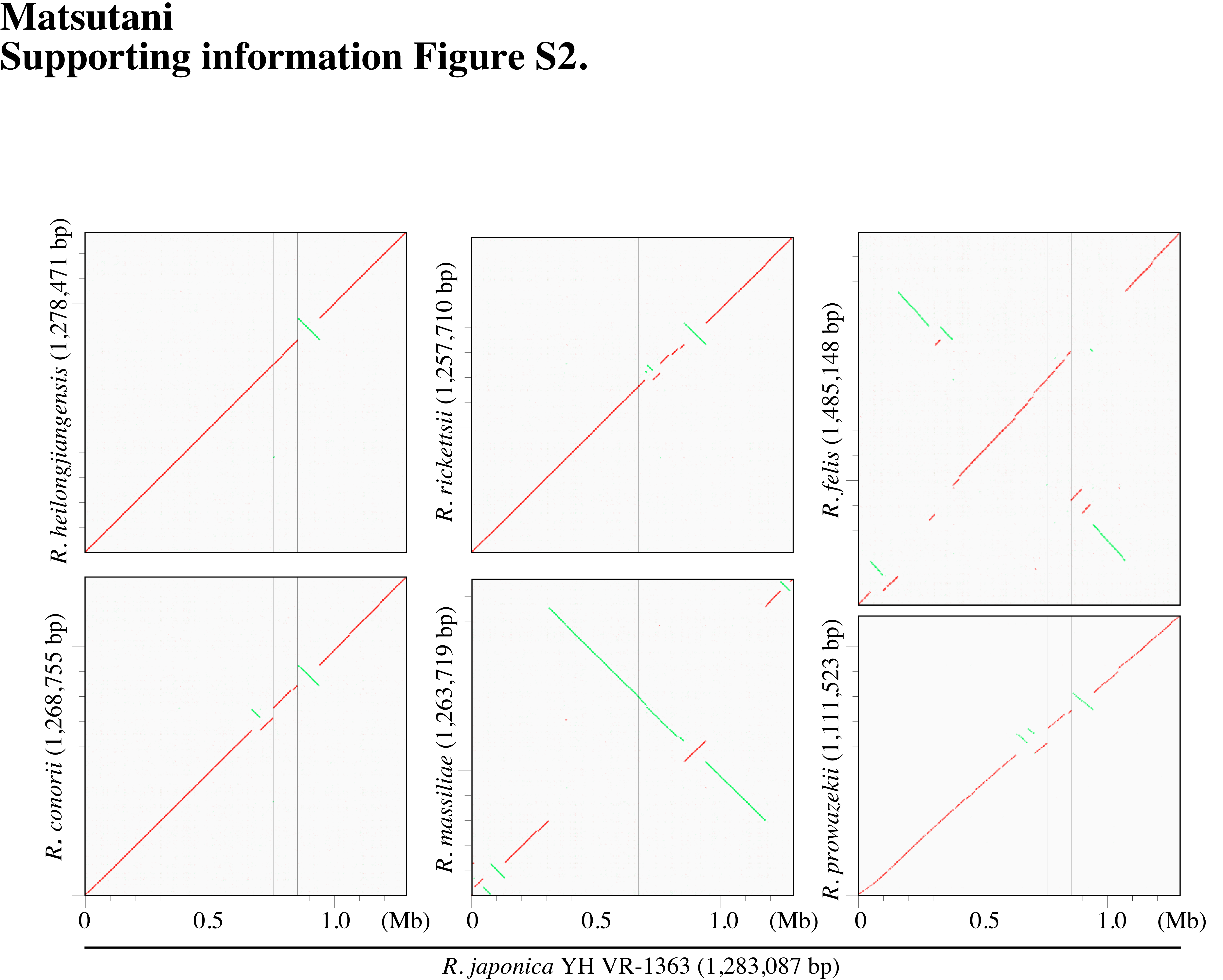

Supplement: Figure S2 — Dot-plot analyses of R. japonica with Rickettsiae. Highly similar regions in genomic DNA sequences of Rickettsia species to R. japonica YH VR-1363 were bi-directionally shown by dot-plot analysis using GenomeMatcher [50]. Red and green spots indicate similar regions in forward- and complementary-strands of each genome sequence, respectively. Inverted and translocation positions were indicated by 4 vertical lines at 668, 754, 851 and 939 kb of R. japonica genome. Rickettsiae used here were R. japonica YH VR-1363 (AP011533), R. conorii Malish 7 (AE006914), R. rickettsii Sheila Smith (CP000848), R. heilongjiangensis 054 (CP002912), R. massiliae MTU5 (CP000683), R. felis URRWXCal2 (CP000053) and R. prowazekii str. Madrid E (AJ235269). (TIF) [file pone.0071861.s002.tif]
