# Supplementary material for: Complete Genomic DNA Sequence of the East Asian Spotted Fever Disease Agent Rickettsia japonica
Source: PLoS One. 2013 Sep 9;8(9):e71861. doi: 10.1371/journal.pone.0071861 (PMC3767692; doi:10.1371/journal.pone.0071861)
Supplement: Table S1 — Rickettsia species for analyses. (DOC) [file pone.0071861.s003.doc]

**Table S1. *Rickettsia* species for analyses**

| **Species** | **Accession number** | **Publication** |
| --- | --- | --- |
| *Rickettsia africae* ESF-5 | CP001612 | Fournier PE, et al., BMC Genomics 10, p166, 2009 |
| *Rickettsia akari* Hartford | CP000847 | Unpublished |
| *Rickettsia australis* Cutlack | CP003338 | Unpublished |
| *Rickettsia bellii* OSU 85-389 | CP000849 | Unpublished |
| *Rickettsia bellii* RML369-C | CP000087 | Gillespie JJ, et al., PLoS Genetics 2, e266, 2007 |
| *Rickettsia canadensis* McKiel | CP000409 | Unpublished |
| *Rickettsia canadensis* str. CA410 | CP003304 | Unpublished |
| *Rickettsia conorii* Malish 7 | AE006914 | Ogata H, et al., Science 293, p2093-8, 2001 |
| *Rickettsia* endosymbiont of Ixodes scapularis | CM000770 | Unpublished |
| *Rickettsia felis* URRWXCal2 | CP000053 | Ogata H, et al., PLoS Biology 3, e248, 2005 |
| *Rickettsia heilongjiangensis* 054 | CP002912 | Duan C, et al., J Bacteriology 193, p5564-5, 2011 |
| *Rickettsia helvetica* C9P9 | CM001467 | Dong X, et al., J Bacteriology 194, p2751, 2012 |
| *Rickettsia japonica* YH VR-1363 | AP011533 | This work |
| *Rickettsia japonica* YH VR-1336 | AMRT00000000 | Dong X, et al., J Bacteriology 194, p6992, 2012 |
| *Rickettsia massiliae* MTU5 | CP000683 | Blanc G, et al., Genome Research 17, 1657-64, 2007 |
| *Rickettsia massiliae* str. AZT80 | CP003319 | Unpublished |
| *Rickettsia montanensis* OSU 85-930 | CP003340 | Unpublished |
| *Rickettsia parkeri* Portsmouth | CP003341 | Unpublished |
| *Rickettsia peacockii* Rustic | CP001227 | Felsheim RF, et al., PLoS ONE 4, e8361, 2009 |
| *Rickettsia philipii* 364D | CP003308 | Unpublished |
| *Rickettsia prowazekii* Madrid E | AJ235269 | Andersson SG, et al., Nature 396,p133-40, 1998 |
| *Rickettsia rhipicephali* 3-7-female6-CWPP | CP003342 | Unpublished |
| *Rickettsia rickettsii* Iowa | CP000766 | Ellison DW, et al., Infect. Immunity 76, p542-5, 2008 |
| *Rickettsia rickettsii* Sheila Smith | CP000848 | Ellison DW, et al., Infect. Immunity 76, p542-5, 2008 |
| *Rickettsia sibirica* 246 | AABW00000000 | Malek JA, et al., Nucleic Acids Research 32, p1059-64, 2004 |
| *Rickettsia slovaca* 13-B | CP002428 | Fournier PE, et al., J Bacteriology 194, p1612, 2012 |
| *Rickettsia typhi* Wilmington | AE017197 | McLeod MP, et al., J Bacteriology 186, p5842-55, 2004 |
| *Candidatus Rickettsia amblyommii* | CP003334 | Unpublished |
